# Editorial: Circadian rhythm in developmental endocrinology

**DOI:** 10.3389/fendo.2023.1210720

**Published:** 2023-05-31

**Authors:** Ebtesam A. Al-Suhaimi

**Affiliations:** Biology Department, Science College and Institute for Research & Medical Consultations, Imam Abdulrahman Bin Faisal University, Dammam, Saudi Arabia

**Keywords:** circadian, rhythm, maternal, developmental, fetus, endocrinology

Physiological functions are regulated by the biological clock, including endocrine functions. The biological clock is related to the astonishing system of “Circadian rhythm” that permits humans to acclimate to alternative changes. Most life forms on our planet have endogenous circadian clocks to adjust to 24-h cycles in conservational stresses compelled by the rotation of the earth around its axis. Humans interact with environmental changes throughout the life span, as circadian clock maturation takes place in humans and experimental animals in embryonic phases, after birth, and improves during puberty and adolescence as well as with age advancement. The impact of circadian rhythm regulation on reproductive systems plays a prominent role throughout the life course (1) Circadian rhythm adjusts energy metabolism, inflammatory processes, cellular renewal, and the interplay with the gut microbiota. Furthermore, circadian rhythm has impacts on gene expression and organ remodeling in fetal development. Therefore, understanding the links between circadian rhythm in pregnancy and fetal development requires insights not just for circadian physiology but also a novel strategy for preventive care for a successful pregnancy and perfect development of the fetus. This Research Topic has been edited and published in four research papers: two original articles and two reviews. During the course of gestation, the hormonal system responds to the demands of the developing fetus. There are maternal signals that are able to synchronize fetal circadian rhythms and vice versa. Bates and Herzog (2) identified five criteria that fulfill the role of this synchronization: hormonal factors, which happened in a circadian manner throughout the pregnancy course; signals derived from the mother or the fetus only; complementary receptor distribution; and being eligible for crossing the placenta. Main maternal hormones synchronize rhythms in the fetus in a daily manner. Therefore, any disturbance in the maturation of the fetal circadian systems exposes the offspring to a higher risk of diseases such as obesity and cardiovascular disease (3, 4). Hormones such as melatonin, glucocorticoids, and dopamine meet the criteria for synchronizing the offspring. Thus, hormones that are exogenously given in pregnancy must be given full attention to their natural diurnal rhythm (2). The light rhythm in the maternal phase is one of the most effective regulators of the fetus. In this Research Topic, Halabi et al. investigated the effect of gestational chronodisruption on the upcoming male rat offspring; this effect weakens physiological processes of glucose homeostasis and adipose tissues. They applied a model of chronic photoperiod alteration during gestation in Sprague-Dawley strain pregnant female rats that have been exposed to lighting manipulation. In the offspring, it was observed that there was an increase in body weight, glucose, adipose tissue amount as a response to norepinephrine (NE), and and changes in the adipose tissue proteome. In an *in vitro* study by Halabi et al., the glycerol response to NE from the same offspring tissues was decreased. At the proteomic level in offspring, 275 proteins showed several changes in expression, including 20 common proteins (2 upregulated and 18 downregulated), and it was found that two functional pathways (AKT/ERK) and (TNF/IL4) were markedly changed by chronic photoperiod alteration during gestation. The study supported the concept that adipose tissue physiology is programmed *in utero* by gestational chronodisruption, leading to metabolic alterations that continue into adulthood. Interestingly, biodynamic lighting status maintains nocturnal melatonin release in hospitalized pregnant women. Bagci et al. found that the maternal circadian system harmonizes with the clock of the fetus under environmental light and dark changes through the melatonin pulse. It was reported that the dramatically changed lighting conditions of hospitalized pregnant women can be improved by installing biodynamic lighting systems in the patient rooms. Jiang et al. found that circadian rhythm is affected by transcription factor BMAL1 in the reproductive system. Aryl-hydrocarbon receptor nuclear translocator like protein 1 (BMAL1), found in the brain and muscle, is a core factor of circadian oscillation. BMAL1-knockout (KO) mice were infertile, had a weak reproductive system, and had the hypothalamus-pituitary-gonadal axis disrupted. Additionally, both BMAL1-KO mice and BMAL1-knockdown by small interfering RNA (siRNA) were studied *in vitro* in steroidogenic cell culture and showed that BMAL1 was connected with steroidogenesis and the expression of related genes in gonads. Furthermore, BMAL1 contributes to the pathogenesis of the human reproductive system. There is deterioration of reproductive components and related endocrine hormones in BMAL1-KO mice. Furthermore, circadian hormone secretion by enteroendocrine cells is a vital function in physiology and in pregnancy. Food intake enhances many incretin, which are produced by gut enteroendocrine cells in a circadian rhythm. This action not only acts locally but also stimulates insulin release in the circulation. Pregnancy is linked with an increase in beta-cells, overweight, and the chance of gestational diabetes mellitus. Timing of diet intake is a recommended strategy to manage metabolic distributions through pregnancy. Homeida et al. focus on the circadian rhythms and physiological functions of enteroendocrine hormones during pregnancy. Food intake and the intestines circadian rhythms regulate the release of enteroendocrine peptide hormones in pregnancy. This Research Topic highlighted the importance of the following: 1) The light rhythm in the maternal phase is an effective regulator of glucose and adipose tissue metabolism in the fetus. 2) In gestational chronodisruption, the glycerol response to NE from offspring tissues decreased, which could be attributed to changes in both pathways (AKT/ERK) and (TNF/IL4). 3) In hospitalized pregnant women, biodynamic lighting conditions maintain nocturnal melatonin release. 4) The circadian rhythm is markedly affected by the transcription factor BMAL1, which has crucial functions in reproductive endocrinology and fertility for steroidogenesis and the expression of related genes in gonads. 5) Circadian rhythms contribute to enteroendocrine hormones during gestation. This highlights a key strategy for better fetal development and circadian entrainment and the prevention of preterm birth. Daily rhythms of maternal, fetus, and placenta signals are attracting research with immense prospects for translation into clinical attention for the best possible gestation and fetus.

## Author contributions

The author confirms being the sole contributor of this work and has approved it for publication.
